# Effect of Phase-Change Nanodroplets and Ultrasound on Blood–Brain Barrier Permeability In Vitro

**DOI:** 10.3390/pharmaceutics16010051

**Published:** 2023-12-28

**Authors:** Stavros Vlatakis, Weiqi Zhang, Sarah Thomas, Paul Cressey, Alexandru Corneliu Moldovan, Hilde Metzger, Paul Prentice, Sandy Cochran, Maya Thanou

**Affiliations:** 1 Institute of Pharmaceutical Science, King’s College London, London SE1 9NH, UK; k1927326@kcl.ac.uk (S.V.); weiqi.zhang@kcl.ac.uk (W.Z.); sarah.thomas@kcl.ac.uk (S.T.); paul.cressey@kcl.ac.uk (P.C.); 2 James Watt School of Engineering, University of Glasgow, Glasgow G12 8QQ, UK; alexandru.moldovan@glasgow.ac.uk (A.C.M.); h.metzger.1@research.gla.ac.uk (H.M.); paul.prentice@glasgow.ac.uk (P.P.); sandy.cochran@glasgow.ac.uk (S.C.)

**Keywords:** nanodroplets, ultrasound, blood-brain barrier, drug delivery, permeability

## Abstract

Phase-change nanodroplets (PCND;NDs) are emulsions with a perfluorocarbon (PFC) core that undergo acoustic vaporisation as a response to ultrasound (US). Nanodroplets change to microbubbles and cavitate while under the effect of US. This cavitation can apply forces on cell connections in biological barrier membranes, such as the blood–brain barrier (BBB), and trigger a transient and reversible increased permeability to molecules and matter. This study aims to present the preparation of lipid-based NDs and investigate their effects on the brain endothelial cell barrier in vitro. The NDs were prepared using the thin-film hydration method, followed by the PFC addition. They were characterised for size, cavitation (using a high-speed camera), and PFC encapsulation (using FTIR). The bEnd.3 (mouse brain endothelial) cells were seeded onto transwell inserts. Fluorescein with NDs and/or microbubbles were applied on the bEND3 cells and the effect of US on fluorescein permeability was measured. The Live/Dead assay was used to assess the BBB integrity after the treatments. Size and PFC content analysis indicated that the NDs were stable while stored. High-speed camera imaging confirmed that the NDs cavitate after US exposure of 0.12 MPa. The BBB cell model experiments revealed a 4-fold increase in cell membrane permeation after the combined application of US and NDs. The Live/Dead assay results indicated damage to the BBB membrane integrity, but this damage was less when compared to the one caused by microbubbles. This in vitro study shows that nanodroplets have the potential to cause BBB opening in a similar manner to microbubbles. Both cavitation agents caused damage on the endothelial cells. It appears that NDs cause less cell damage compared to microbubbles.

## 1. Introduction

Glioblastoma multiforme (GBM) stands out as one of the most aggressive and common types of brain tumors, marked by a bleak prognosis. The average survival rate for GBM patients typically falls within the range of 12–18 months, with an incidence rate spanning from 0.59 to 3.69 per 100,000 individuals. [1]. Following tumour surgical removal and radiation therapy, chemotherapeutic drugs are used for the suppression of the disease. However, apart from the aggressiveness and high recurrence of GBM, the blood–brain barrier (BBB) and the blood–brain tumour barrier (BBTB) minimize the chemotherapy’s effectiveness. These two barriers block the delivery of the drugs, making it hard for the medications to reach the therapeutic concentration at the tumour site. Therefore, there is a lot of scientific interest in the BBB opening to enhance drug delivery in GBM tumours [2].

Recently, the combination of focused ultrasound (FUS) and microbubbles (MBs) showed promising pre-clinical and clinical data for a safe, controlled, and reversible BBB opening. Image-guided FUS offers a local and non-invasive intervention that can target specific brain areas without damaging the skull and neighboring tissues [3,4]. MBs are spherical gas-filled microparticles (size 1–10 μm) that consist of a shell, usually made of a lipid assembly, and a perfluorocarbon (PFC) core. After intravenous MB injection and local FUS irradiation, the MBs start cavitating (expand and contract) and provide a controlled, localized motion. This applies forces that break the cell-to-cell contact that temporarily disrupts the BBB [5,6].

However, MBs’ size and composition lead to limited circulation time in vivo, as they rapidly decompose and are cleared by the liver (after a few minutes) [5]. Therefore, to overcome these limitations, the development of perfluorocarbon nanoemulsions, also known as phase-changed nanodroplets (NDs), has been a field of significant interest for the last two decades [7]. NDs comprise a lipid or polymeric shell enveloping a condensed PFC gas core [7]. The super-heated condensed gas core enables them to circulate in the bloodstream in a bioinert state, which is facilitated by the provided Laplace pressure. The PFC gas is inert and is excreted through the lungs [8]. The NDs’ small size (100–150 nm) also gives them the ability to potentially infiltrate through the endothelial gaps concentrated in tumour sites, and they have been shown to have far superior circulation times up to 2–6 h [9]. After the targeted US application, NDs’ PFC vaporizes and transforms into MBs under the acoustic droplet vaporization (ADV) effect [10]. During ADV, the applied US shifts the vapor pressure equilibrium of the saturated PFC liquid into its vaporized form, leading to MB generation and, therefore, cavitation and BBB opening [11]. Lastly, the ADV properties of NDs can be modified by changing the type of PFC encapsulated or the lipid shell of the formulation [12,13].

Phase-change NDs represent an innovative approach to drug delivery, offering distinct advantages over microbubbles [7]. The preparation methods for these nanodroplets involve novel techniques and utilize diverse materials as shells, combined with different PFCs. This study addresses a critical need for comprehensive and holistic nanodroplet characterization, encompassing both their solution properties ADV and cavitation, as well as their interaction with cells during these events. The novelty of our study lies in the unique method employed for the ND preparation, emphasizing the exploration of their physicochemical properties and in vitro cell assays to understand their effect on endothelial barriers. By focusing on the formulation and characterization of perfluorocarbon core NDs, our research aims to provide a deeper understanding of their behaviour and performance. One key aspect of our investigation involves the assessment of in vitro BBB model permeability. By combining US with NDs, we seek to elucidate the synergistic effects and potential enhancements in the permeability of the endothelial and their promising applications in drug delivery to the brain.

In summary, this study contributes to the advancement of nanodroplet technology but also addresses the specific advantages they can offer over conventional microbubbles.

## 2. Materials and Methods

1,2-Dipalmitoyl-sn-glycero-3-phosphocholine (DPPC; 16:0 PC) and (ω-methoxy-polyethyleneglycol2000)-N-carboxy-1,2-distearoyl-sn-glycero-143-phosphoethanolamine (DSPE-PEG_2000_-OMe) were purchased from Avanti Polar Lipids (Alabaster, AL, USA). Perfluoropentane (PFP), perfluorohexane (PFH), chloroform, methanol (MeOH), glucose, HEPES, fluorescein, Dulbecco’s Modified Eagle’s Medium (DMEM), Trypsin–EDTA solution, and PBS were purchased from Sigma Aldrich (Gillingham, UK). For the DSC and DLS, Nano DSC from TA Instruments (New Castle, DE, USA) and Malver Zetasizer Nano S (Malvern, UK) were used, respectively. LIVE/DEAD™ Cell Imaging Kit (488/570) was purchased by Invitrogen (Massachusetts, USA) and 12-well Thinsert^TM^ (0.4 μm pore size, PET membrane) from Greiner bio-one (Stonehouse UK). For the MB experiments, we used Sonazoid^TM^ (GE healthcare, Illinois USA) and SonoVue^®^ (Bracco Imaging S.p.A., Milan Italy).

### 2.1. Nanodroplet Fabrication

The nanodroplets (15 mg/mL lipid concentration) were prepared at the following 93:7 DPPC: DSPE–PEG_2000_ mol% ratio. The calculated amount of DPPC and DSPE-PEG_2000_ was added into a round bottom flask. The solvent was then removed in vacuo to form a thin film. The film was left under vacuum for at least 24 h to remove any residual organic solvents. A buffer solution (20 mM HEPES with 10% *w*/*v* glucose pH 7.4) was added to hydrate the film, and the solution was sonicated in 50–55 °C until a suspension was formed. PFC (1% *v*/*v*) was added to the lipid suspension and then sonicated in an ice bath until a clear nanodroplet solution (nanodroplets dispersed in the buffer) was observed. The ND solution was centrifuged at 5000 rpm for five minutes at 4 °C to remove any unencapsulated PFC, and the supernatant was isolated and stored into a small glass vial at 4 °C.

### 2.2. Dynamic Light Scattering (DLS)

To measure the NDs’ size, the sample was loaded in Malvern Zetasizer at 0.75 mg/mL of ND estimated lipid concentration (1/20 dilution); 25 °C with 30 s settle; three different samples were used with three repeat measurements. The samples were NDs of the same lipid composition but with different core content of 100% PFP and 100% PFH.

### 2.3. Fourier–Transform Infrared Spectroscopy (FTIR)

We employed FTIR analysis to examine the PFC content, following the methodology previously described [14]. Specifically, 20 µL of each sample were introduced into a liquid cell before conducting measurements using the FTIR spectrometer (Tensor II, Bruker Optics, Billerica, MA, United States). The measurements were obtained with a spectral resolution of 8 cm^−1^ and covered a range of 4000–800 cm^−1^ over 16 scans. Additionally, the spectral range of 1000–4000 cm^−1^ was considered during the analysis.

### 2.4. HIFU-Induced Gas Evolution and Cavitation Monitoring with High-Speed Camera (HSC)

**Figure 1:**
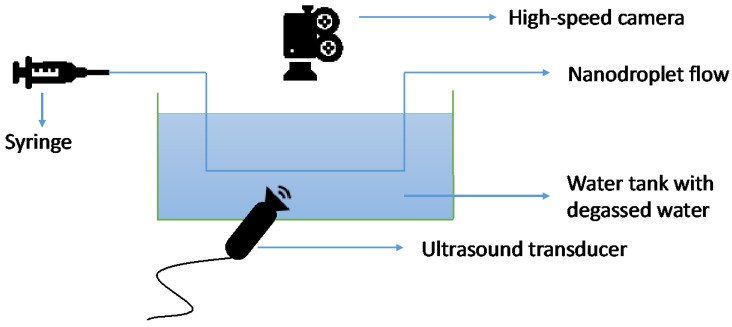
Graphical representation of the HSC setup for the NDs’ cavitation observation after the US activation.

In this experimental setup (Figure 1), the observations were conducted within a custom-designed cavitation tank with dimensions of 420 × 438 × 220 mm^3^, which was filled with de-ionized and degassed water. To generate ultrasound, we utilized a 90 mm diameter transducer (H-198, Sonic Concepts, Bothell, WA, USA) that was excited by a power amplifier (1040L, E&I) and driven for 100 cycles at a frequency of 1.1 MHz using an arbitrary waveform generator (DG4102, Rigol, Beijing, China). The output of the transducer was characterized using a needle hydrophone (Figure S1, Supplementary Materials). Within the central region of the ultrasound focus, we positioned a (0.5 mm inner diameter and 0.7 mm outer diameter) polycarbonate capillary (Paradigm Optics, Vancouver, WA, USA) using a bespoke 3D-printed mount. The inlet and outlet of the capillary were securely connected to silicon tubing using epoxy. To introduce nanodroplets (NDs) into the system, we diluted them approximately 50-fold in degassed water. The NDs were then introduced into the capillary via a syringe equipped with a 20 G microlance, which was inserted into the silicon tubing inlet. The outlet of the silicon tubing was connected to a collection reservoir located outside the tank. The US application was conducted at room temperature. We recorded cavitation dynamics at a high frame rate of 10 × 10^6^ frames per second (fps) over a duration of 25.5 µs using a high-speed camera (HPV-X, Shimadzu, Kyoto Japan) and a 5× objective lens (final resolution ~3 µm/pixel, field of view 400 × 250 pixels). The camera triggering was synchronized with the experiment and initiated 51.6 µs after exciting the transducer to allow the ultrasound waves to propagate from the transducer to the capillary. For illumination purposes, we directed light from below the experimental setup using a liquid light guide and synchronous 10 ns laser pulses generated by a CAVILUX laser system (Cavitar, Tampere, Finland). The HSC setup is depicted in Figure 1.

### 2.5. Blood–Brain Barrier (BBB) In Vitro Model

**Figure 2:**
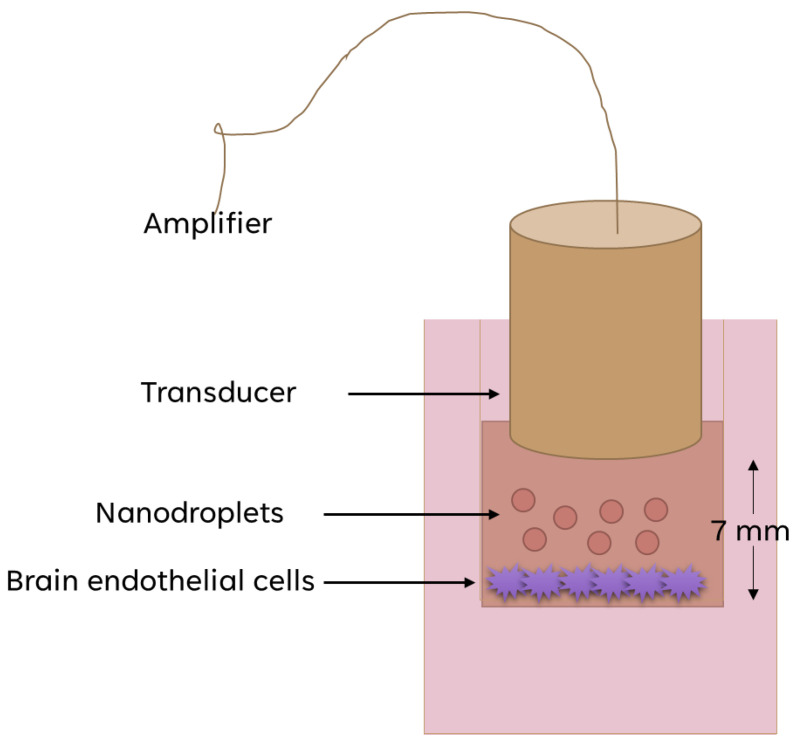
Graphical representation of the in vitro BBB model used for the permeability assessment studies.

The cell line used for the BBB model was b.End.3, ATCC^®^ CRL2299™, Manassas, VA, USA (endothelial cells isolated from brain tissue derived from a mouse with endothelioma) cell line (passage numbers 30–35). The cells were trypsinized when they reached >95% confluency in the flask and were added into the 12-well Thinsert^TM^ (0.4 μm pore size, PET membrane), concentration of 8 × 10^4^ cells per thinsert. After 7 days inside the incubator (37 °C, 5% CO_2_), the cells reached confluency as assessed by light microscopy. The medium was aspirated and replaced with degassed PBS and NDs or SonoVue^®^ MBs. The lipid concentration of both NDs and MBs was around 6.5 mg/mL inside the thinserts. Afterward, ultrasound was applied for 7 min using the 1 MHz octagonal transducer (Figure 2; Figures S2–S5 and Table S1, Supplementary Materials) from 50% duty cycle and 0.66 MPa of pressure amplitude. After the US application, the apical solution was aspirated and replaced with 1 mg/mL sodium fluorescein solution and DMEM. The US application was conducted at room temperature. The cells were incubated at 37 °C for 1 h under mild shaking, and then the permeation of sodium fluorescein was measured (peak excitation at 494 nm and peak emission at 512 nm) using a plate reader. For the control experiments, we left the transwells after adding PBS for 7 min with or without applying US. Then we added the sodium fluorescein, incubated at 37 °C for 1 h under mild shaking, and measured the permeability using the plate reader. The BBB model setup is depicted in Figure 2.

To visualize the effect of the US and ND or US and MB on the viability of BBB cells, we used the LIVE/DEAD cell imaging kit (LIVE/DEAD™ Cell Imaging Kit 488/570-Catalog number: R37601). The reagent was added to the thinserts and incubated for 30 min. The plates were then observed under the fluorescent microscope (Nikon Eclipse Ts2R interted research microscope).

The unfocused transducer used in this study was fabricated using piezocomposite material based on a piezoelectric soft ceramic, PZT–5H (Ferroperm PZ29, CTS Ferroperm, Kvistgaard, Denmark), and a hard-set epoxy (Epofix, Struers Inc., Cleveland, OH, USA). The active element was cut in an octagonal shape, had a resonance frequency of 1.0 MHz, and its US radiating area was 38.4 mm^2^. The backing material consisted of Epofix filled with air-filled micro-balloons. The electrodes on the back and front faces of the transducer were applied via spin coating of a Silver-based ink (118-09A/B119-44, Creative Materials, Ayer, MA, USA). The device was powered by a signal generator (RIGOL Beijing, China) coupled to a power amplifier (RF Power Amplifier, Electronics and Innovation, Rochester, NY, USA) with a 50 dB gain. The output of the transducer was characterized using a needle hydrophone (NH) and a radiation force balance (Figures S2 and S3). The area of the acoustic beam corresponding to the −3 dB pressure profile, measured at 7 mm in front of the transducer, was 5.3 mm^2^. (Figures S4 and S5 and Table S1, Supplementary Materials).

## 3. Results and Discussion

### 3.1. Nanodroplet Preparation and Size Characterisation

In this study, we developed a simple method to prepare NDs, composed of a lipid shell, that is similar to liposome lipid assembly. In this study, we prepared nanodroplets following the adaptation of a recently presented method [14]. Here, we changed the method to include a 10 min treatment of sonication at 55 °C, followed by PFC rapid addition and a 20 min treatment of the vial on the ice bath while it was sonicated. This method yields NDs with an average ND size of around 100 nm. In the method presented here, the dried lipid film (93:7 mol% ratio of DPPC to DSPE–PEG_2000_) is initially hydrated with a low salt concentration buffer solution and hot sonicated until the formation of an opalescent solution. The appearance of the different stages of the solution during the process is critical. The opalescent solution needs to be cooled down before 1% *v*/*v* of PFC solution is added on ice. In this process, avoiding the vaporization of the PFC is emphasized. This step appears critical as PFC is required to be in a liquid state to better associate with the lipids and replace water from the core. In addition, in the method presented in this paper, we have introduced a final step of centrifugation to remove the PFC that is not encapsulated in the lipid shell. The removal of the PFC allows for a better dispersion of the nanodroplets (transparent solution). After this step, and to avoid gas expansion and PFC escape, the vials are stored in the fridge.

During the last several years, several methods of ND preparation have appeared in the literature [7]. Several reports propose the use of microfluidics [11,15]. In our present study, we adapted a liposome preparation method that yielded nano-size droplets significantly smaller to the ones prepared with microfluidics [11,16]. The method we have used appears to be easy to adapt and potentially easy to scale to large quantities as it is a liposome preparation method adaptation.

The ND size remained similar after a week of storage, showing a slight decrease (Table 1 and Figure 2). It is critical to monitor size and PFC content during storage to assess the integrity of the NDs, which will affect their ADV properties.

The PFC core is the most crucial part of the NDs concerning the cavitation profile [17,18]. Different PFC cores are expected to attribute distinct characteristics to the ADV of the NDs [11,15,19]. The differences in the carbon chain, fluorine atoms, and boiling points have a significant role in ADV, cavitation, and, potentially, colloidal stability in biological fluids [20]. The stability of PFC NDs in biological fluids is essential as NDs need to contain the suitable PFC amount when reaching the BBB. In other words, PFC should not leak out of the NDs.

**Figure 3:**
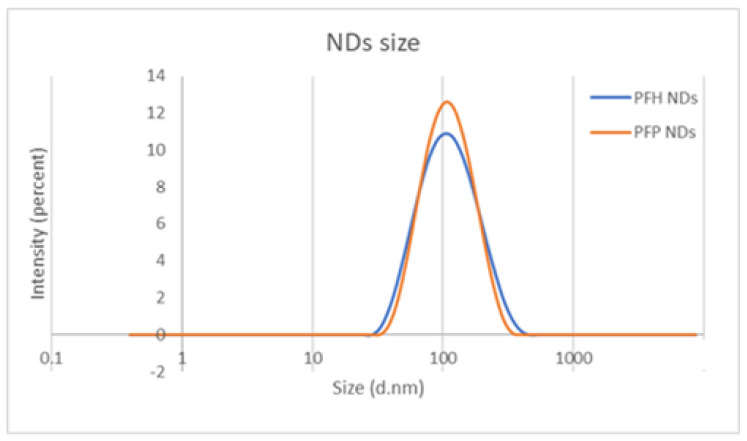
Effect of different PFC cores on the nanodroplet size as measured by dynamic light scattering.

In this study, we have formulated NDs with three different PFC cores: perfluoropentane (PFP), perfluorohexane (PFH), and a 1–1 mixture of PFP:PFH (1:1 volumetric ratio). PFP has a boiling point of 29 °C, while PFH has a boiling point of 56 °C. The PFC type did not affect the resulting average ND size, which appeared similar and in agreement with previous studies [21] (Figure 3).

### 3.2. Using FTIR to Quantify PFC Content

**Figure 4:**
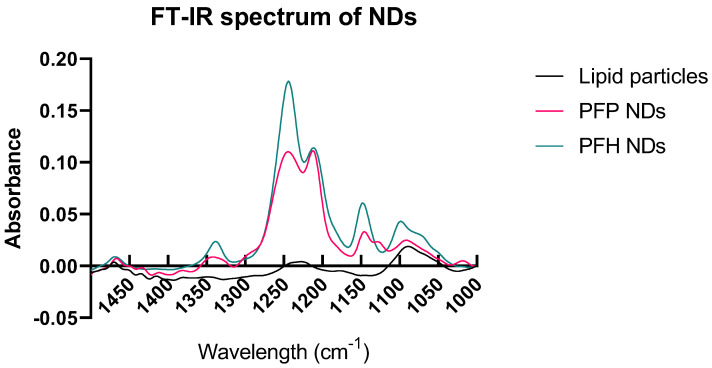
FTIR spectrum of NDs and lipid particles. The existence of PFC is evident with the two major peaks between 1300–1200 cm^−1^ in both PFH and PFP NDs.

FTIR is a widely used spectroscopy analytical technique. In our study, it is used to detect the signals of the encapsulated nanodroplet PFC chemical bonds. Following the study of Choi et al., and our previously reported method [14], we designed a method of FTIR to detect the PFC encapsulation in the ND core [22]. Initially, the FTIR spectrum of the lipid NDs before adding PFC showed no significant peaks between 1500–1000 wavenumbers (cm^−1^) (Figure 4). However, after the ND formation with the PFC addition, we observe a double peak between wavelengths 1300–1200 cm^−1^. The peaks between 1300–1200 cm^−1^ are characteristics of the C–F bond and indicative of the PFC content [22]. The two different PFC cores tested provided similar spectra. The main difference is that the PFH NDs showed a higher peak because of the extra C–F bonds, as expected. It appears that FTIR provides a quick and efficient technique to prove the PFC encapsulation after the ND formation. Characterizing the content of the nanodroplets is of high importance when a novel method of ND preparation is introduced.

### 3.3. High-Speed Camera Capture of ADV and Cavitation Nanodroplets

**Figure 5:**
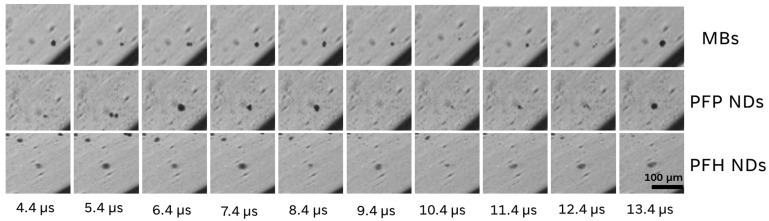
Individual HSC frames after US application to observe the cavitation profile of MBs and different PFC-core NDs.

One of the most important phenomena of the combination of US and gas-containing particles is cavitation. Imaging such phenomena can provide significant information regarding the nanodroplet or microbubble reaction to US [23,24]. After we successfully formulated and characterized the NDs, we investigated their cavitation. For these experiments, we used the HSC camera method to observe the expansion and contraction of the NDs after the US activation. To ensure the tube was free of bubbles, degassed buffer was used as a negative control and showed zero bubble cavitation throughout the HSC frames. Similar frames and videos were recorded observing the cavitation of Sonazoid™ MBs activated under equivalent US conditions of the NDs (Videos S1–S3, Supplementary Materials). The main observation is that MBs and NDs have almost identical cavitation profiles after the US application in various pressure amplitudes, sustaining the proof that the prepared NDs can undergo ADV and cavitate Figure 5 shows the individual frames during the MB and ND cavitation events at 0.600 MPa peak-negative pressure (PNP). Comparing the cavitation profiles in various PNP values (0.120 to 1.2 MPa), we can observe that the bubble fragmentation is faster as it was expected, while the pressure amplitude increases (videos in Videos S1–S3). The different PFC-core NDs (PFP, PFH) did not show significant differences in terms of cavitation observations. In our previous data, similar PFC core nanodroplets did not show a difference in cavitation profiles when different PFC cores were used [14]. The lowest threshold we managed to observe in ND cavitation was at 0.120 MPa (Video S4, Supplementary Materials). All of the experiments were conducted at room temperature. Summarizing the observations above, we show here that our novel NDs can cavitate after US application, providing a similar cavitation profile to the Sonazoid^®^ MBs. It will be interesting to investigate if the ADV threshold affects the NDs’ cavitation profile [25,26]. Such information can substantially improve the design of nanodroplets for imaging and therapy. The components composing the shell and the core are expected to play a significant role in ADV and cavitation mode.

### 3.4. Effect of US and NDs on Blood–Brain Barrier (BBB) Permeability In Vitro

**Figure 6:**
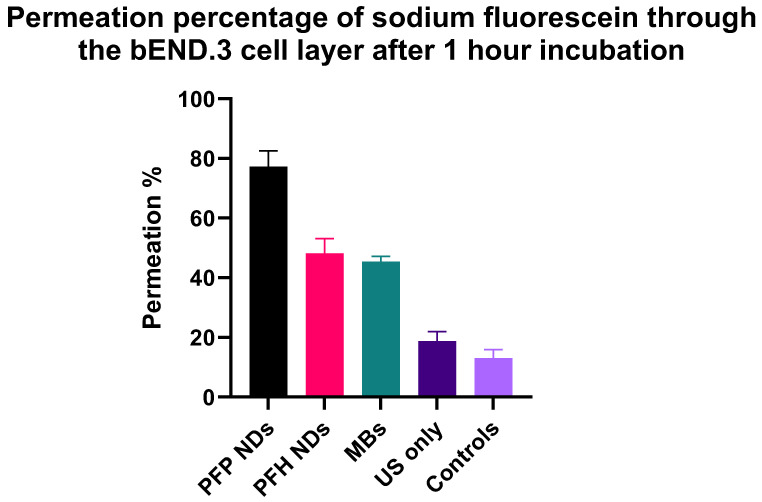
Effect of US and cavitation agents on fluorescein permeability across BBB bend, 3 cells in vitro. US was applied for 7 min using 1 MHz transducer, 50% duty cycle, and 0.66 MPa pressure amplitude. Permeability of fluorescein was assessed at 1 h post-US application (*n* = 3, mean ± SD).

The FUS–MB combination can cause BBB opening, which has been extensively reported [3,27,28]. Most of the studies that report enhanced BBB permeability use microbubbles as cavitation agents. However, there is a small number of studies that present the effect of the combination of FUS and nanodroplets on BBB opening [29,30,31]. Most of the studies on the BBB FUS opening present the effects in vivo, which significantly limits the development of novel cavitating agents. There is a lack of suitable in vitro experimental set-ups that would help us understand the effect of cavitation and design suitable cavitating agents. There are two main studies that assess the permeability of an in vitro endothelial cell model using the US and cavitation agents’ effects on cells. Shen Y. et al. prepared a b.END3 cell monolayer model to assess the permeability after the combination of focused ultrasound and MBs using sodium fluorescein as a permeating molecule [32]. Fix S. et al. prepared a Caco-2 (colorectal adenocarcinoma cells) to mimic the intestinal epithelial cell barrier, and they assessed the permeability after the combination of US and polymer shell NDs using a hydrophilic macromolecule fluorescein isothiocyanate (FITC)–dextran (70 kDa) [33]. In our work, we constructed an in vitro b.End3 cell model, which is widely used for BBB permeability studies. However, it has never been tested in studying the effects of ultrasound and MB. This model includes the culture of the cells as monolayers on membrane inserts that can support the application of US to cause the cavitation of either MBs or NDs. After the cell membrane reached confluency, we introduced the NDs or MBs onto the apical side and started the US application. The transducer frequency of the handmade planar transducer and PNP were 1 MHz and 0.66 MPa, respectively, similar to the pressure amplitude used for the HSC cavitation observation experiments. These values are in line with the FDA-recommended PNP values to be used in clinical trials [34]. After the US application, the permeability was assessed using fluorescein for 1-h incubation. The fluorescein (with molecular weight 332.31 g/mol) permeability was measured at the basolateral side and (Figure 6) showed a 4-fold permeability increase after the combination of PFP–NDs and US compared to just US application and a 1.7-fold increase comparing the combination of MBs and US. It is possible that the PFP–NDs demonstrate different types of cavitation compared to the PFH–NDs and the MBs. US combined with PFH–NDs effected less permeability increase compared to the PFP–NDs after the US application.

**Figure 7:**
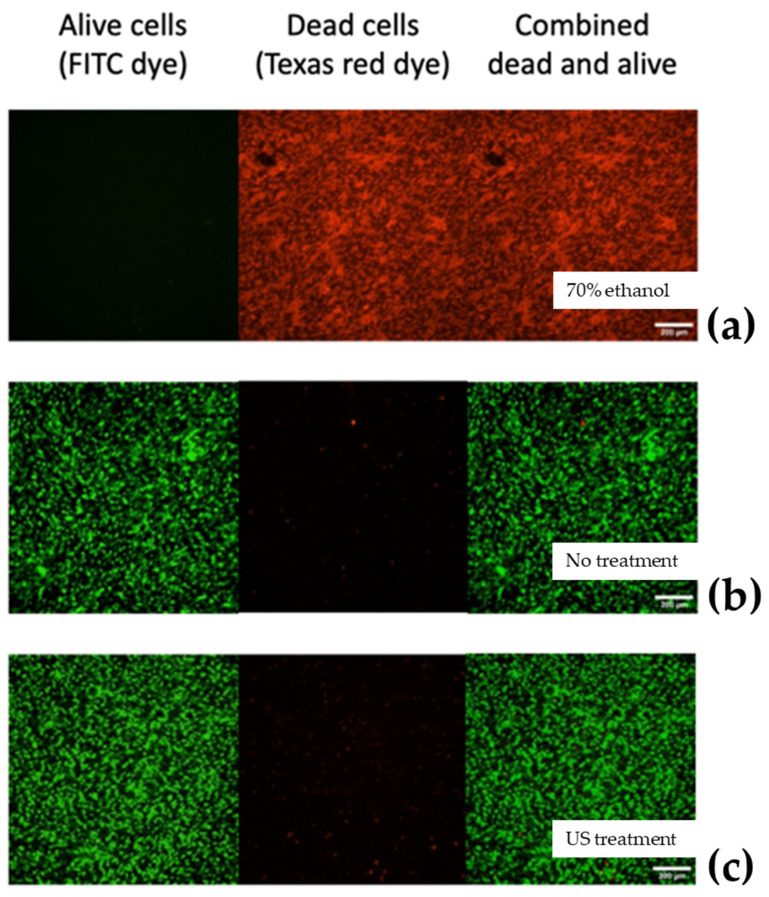
Fluorescent microscope images after the incubation with the LIVE/DEAD imaging kit. In (**a**), the cells were incubated at 70% to visualize the total cell death (the cells were coloured with the Texas Red dye), while (**b**,**c**) show untreated cells and US-treated cells, respectively.

The fluorescein permeability increase may also be due to US cavitation causing cell membrane-damaging effects. To assess endothelial barrier integrity after the combination of cavitation agents NDs and MBs, as well as US, we used the LIVE/DEAD cell-imaging kit and fluorescent microscopy. To allow an image of extreme cell damage, we co-incubated cells with a 70% ethanol solution as this would cause complete cell death (Figure 7a). We observed no difference in the appearance of untreated and US-only treated cells (Figure 7b,c). This indicates that the US alone applied on endothelial cells does not affect the viability of cells.

To assess the cell viability in the US focal region, we utilized the LIVE/DEAD^®^ Cell Imaging Kit. In this kit, live cells were fluorescent green due to the intracellular activation of a cell-permeable calcein dye following the cleavage of esters by intracellular esterases. Conversely, dead cells lose membrane integrity and are, therefore, fluorescent red following DNA binding of the cell impermeable dye.

**Figure 8:**
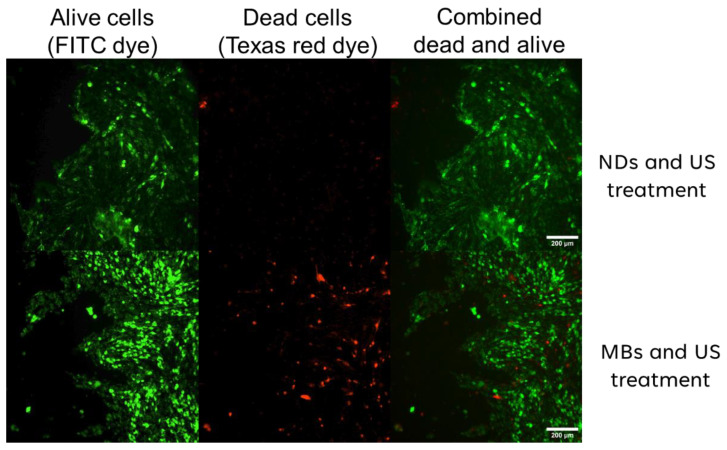
In Figure 8, we observe what the fluorescent microscope images captured following incubation with the LIVE/DEAD imaging kit. The upper panel depicts live, dead, and combined LIVE/DEAD fluorescent images obtained after concurrent US and PFP core ND treatments. The lower panel displays corresponding live, dead, and combined LIVE/DEAD fluorescent images following the application of US and MB treatments.

The synergistic application of ultrasound in conjunction with PFP NDs resulted in a pronounced induction of cell death, accompanied by the formation of breaches within the bEnd.3 cell monolayer, as depicted in Figure 8 (upper panel). In this experimental investigation, we assessed the impact of NDs on cellular permeability across bEnd.3 cell monolayers, highlighting their potential advantages compared to microbubbles. The observed formation of cell monolayer disruptions attests to the compromised integrity of the blood–brain barrier following the application of US in tandem with MBs and/or NDs, potentially leading to enhanced molecular permeability. Figure 8 (upper panel) showcases the combined LIVE/DEAD cell images surrounding the US-sonicated endothelial cell membrane providing evident visual confirmation of both hole formation and cellular demise (see Figure S6 for more fluorescent images).

In the subsequent set of experiments, our focus centered on visualizing cell death in the combination of US and MBs. These investigations unveiled a considerably heightened level of cellular mortality and hole formation, as depicted in Figure 8 (lower panel), (see Figure S7 for more fluorescent images). Notably, the formation of larger breaches, coupled with intensified membrane destabilization, surpassed the effects observed in the US and ND experiments.

Summarizing the LIVE/DEAD cell-imaging kit experiments, we can suggest that the combination of NDs and/or MBs, as well as US, may cause significant damage to the endothelial cell monolayer, which is conveyed via the presence of holes. However, MBs cause more extensive damage and compromise the cell monolayer in a more vigorous way compared to NDs. Therefore, phase-changed nanodroplets could provide a more controlled and less harmful way to open the BBB compared to MBs in the defined US parameters used in these experiments. Nevertheless, further investigation across diverse US conditions is imperative to acquire a broader understanding of the impact of either MBs and/or NDs on the cell monolayer subsequent to the cavitation phenomenon induced by ultrasound.

## 4. Conclusions

In this study, we have prepared nanodroplets and confirmed the encapsulation of PFC as part of the core using FTIR. Furthermore, our observations of the cavitation effect through a high-speed camera provide evidence of this ND cavitation. In order to assess the potential applications of our NDs, we constructed an in vitro BBB model using b.End3 cells. We used a consistent pressure amplitude of 0.66 MPa, thereby corroborating the presence of cavitation events in both NDs and MBs. Through meticulous experimentation involving ND and US applications, we have demonstrated that NDs, in conjunction with US, can compromise the integrity of the cell monolayer, leading to a significantly higher increase in sodium fluorescein permeability compared to MBs and US. The results of our microscopy viability assay reveal cell damage and hole formation within the cell monolayer after the combination of US and NDs, albeit in an apparently less severe manner when compared to the combination of US and MBs. Importantly, our findings indicate that the use of NDs in conjunction with US offers a potentially more controlled approach when compared to traditional MBs. This work has the potential to significantly advance drug delivery and therapeutic strategies for neurovascular disorders. However, we need to aim for the development of a more representative BBB model to have a better understanding and assessment of the in vitro data, as these models can offer a good prediction of the effects of US and cavitation agents on the intact BBB.

## Figures and Tables

**Table 1:** NDs containing different PFC cores. Size was assessed after preparation and after 7 days of storage at 4 °C.

Perfluorocarbon (PFC) Core	Size (nm)	Polydispersity Index (PDI)	Size (nm) 7 Days After	PDI 7 Days After
Perfluoropentane (PFP)	121.3 ± 2.4	0.23 ± 0.0	112.5 ± 2.1	0.21 ± 0.0
Perfluorohexane (PFH)	123.3 ± 0.95	0.2 ± 0.0	106.0 ± 1.58	0.24 ± 0.0

## Data Availability

The data presented in this study are available on request from the corresponding author. The data are not publicly available due to the current use in other studies by the authors that are intended to be published in the near future.

## Supplementary Materials

The following supporting information can be downloaded at: https://www.mdpi.com/article/10.3390/pharmaceutics16010051/s1, Figure S1: Calibration curve for the correlation of the amplitude to pressure values, Figure S2: Calibration curve for the correlation of the amplitude to pressure values, Figure S3: Correlation of mVpp to pressure amplitude using the RFB calibration, Figure S4: The image depicts the XZ scans of the symmetric and asymmetric transducers, Table S1: The table shows the beam average diameter of the transducers after the XZ scan, Figure S5: The figure depicts the intensity spatially averaged over the area enclosed by the half-pressure-maximum contour (ISAL), Figure S6: In this figure we observe various combined images of the well plates where the cell death and hole formation by the ND cavitation compromise the integrity of the BBB model, Figure S7: In this figure we observe various combined images of the well plates where the cell death and hole formation by the MB cavitation compromise the integrity of the BBB model in a more violent way, Video S1: With High-Speed Camera (HSC) imaging, we have observed the cavitation dynamics exhibited by Sonazoid ® microbubbles (MBs) when subjected to ultrasound at a frequency of 1.1 MHz, accompanied by a peak negative pressure of 0.60 MPa. The video illustrates the expansion and subsequent collapse of the MBs in synchrony with the ultrasonic frequency, Video S2: With High-Speed Camera (HSC) imaging, we have observed the cavitation dynamics exhibited by PFP NDs when subjected to ultrasound at a frequency of 1.1 MHz, accompanied by a peak negative pressure of 0.60 MPa. The video illustrates the expansion and subsequent collapse of the NDs in synchrony with the ultrasonic frequency, Video S3: With High-Speed Camera (HSC) imaging, we have observed the cavitation dynamics exhibited by PFH NDs when subjected to ultrasound at a frequency of 1.1 MHz, accompanied by a peak negative pressure of 0.60 MPa. The video illustrates the expansion and subsequent collapse of the NDs in synchrony with the ultrasonic frequency, Video S4: With High-Speed Camera (HSC) imaging, we have observed the cavitation dynamics exhibited by PFP NDs when subjected to ultrasound at a frequency of 1.1 MHz, accompanied by a peak negative pressure of 0.12 MPa. The video illustrates the expansion and subsequent collapse of the NDs in synchrony with the ultrasonic frequency.
