# Supplementary material for: Effect of Phase-Change Nanodroplets and Ultrasound on Blood–Brain Barrier Permeability In Vitro
Source: Pharmaceutics. 2023 Dec 28;16(1):51. doi: 10.3390/pharmaceutics16010051 (PMC10818572; doi:10.3390/pharmaceutics16010051)
Supplement: Supplementary file 1 [file pharmaceutics-16-00051-s001.zip › pharmaceutics-2725739-supplementary.pdf]

Effect of Phase-Change Nanodroplets and Ultrasound on Blood–Brain Barrier Permeability In Vitro

Stavros Vlatakis [1], Weiqi Zhang [1], Sarah Thomas [1], Paul Cressey [1], Alexandru Corneliu Moldovan [2], Hilde Metzger [2], Paul Prentice [2], Sandy Cochran [2] and Maya Thanou [1,*]

[1] Institute of Pharmaceutical Science, King's College London, London SE1 9NH, UK; k1927326@kcl.ac.uk (S.V.); weiqi.zhang@kcl.ac.uk (W.Z.); sarah.thomas@kcl.ac.uk (S.T.); paul.cressey@kcl.ac.uk (P.C.)
[2] James Watt School of Engineering, University of Glasgow, Glasgow G12 8QQ, UK; alexandru.moldovan@glasgow.ac.uk (A.C.M.); h.metzger.1@research.gla.ac.uk (H.M.); paul.prentice@glasgow.ac.uk (P.P.); sandy.cochran@glasgow.ac.uk (S.C.)
* Correspondence: maya.thanou@kcl.ac.uk

90 mm diameter transducer calibration data using needle hydrophone

A scanning tank with degassed water and a 0.2 mm needle hydrophone (Precision Acoustics, Dorchester, UK) were used to calibrate the transducer and correlate the voltage to pressure amplitude (Figure S1).

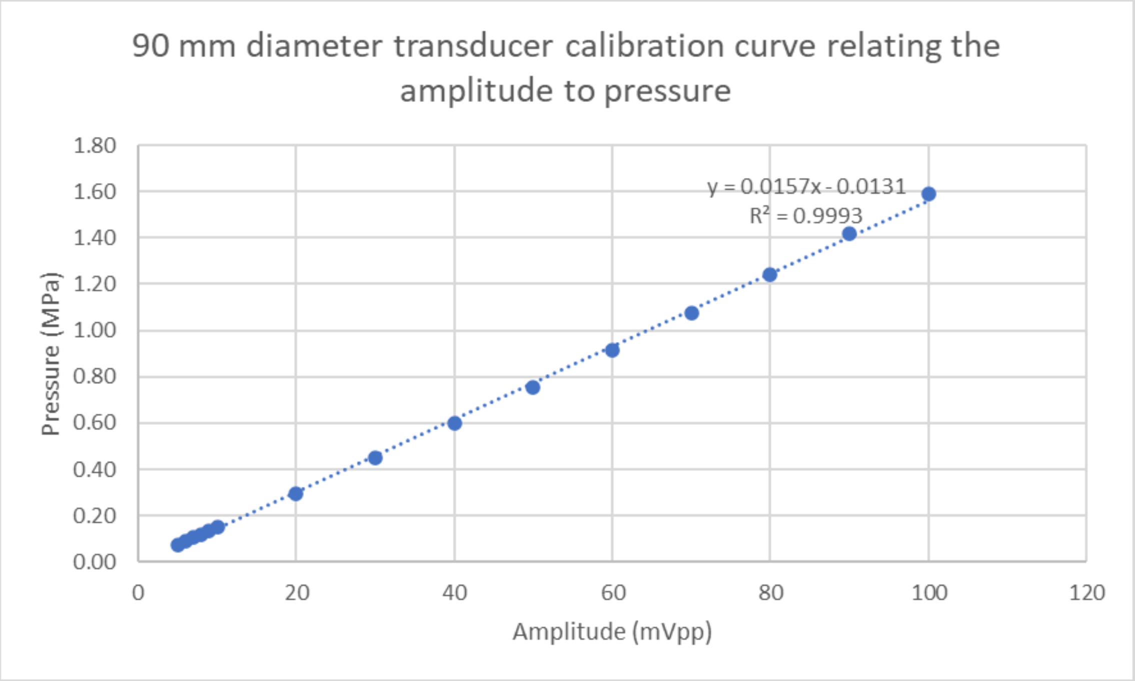

Figure S1: Calibration curve for the correlation of the amplitude to pressure values.

Unfocussed transducer calibration data using needle hydrophone

A scanning tank with degassed water and a 0.2 mm needle hydrophone (Precision Acoustics, Dorchester, UK) were used to calibrate the transducer and correlate the voltage to pressure amplitude (Figure S2).

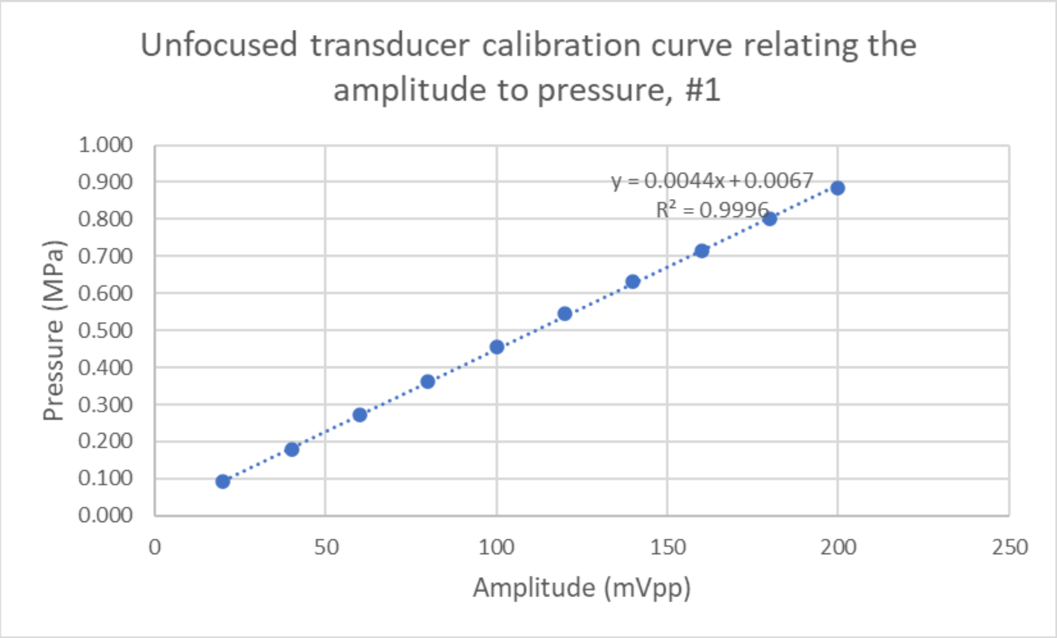

Figure S2: Calibration curve for the correlation of the amplitude to pressure values.

Unfocussed transducer calibration data using radiation force balance (RFB)

Radiation Force Balance Measurements

Acoustic power measurements of the single element transducer were conducted into a tank with deionized, degassed water using an acoustic radiation force balance (RFB – Precision Acoustics, Dorset, United Kingdom). The distance between the transducer and the acoustic absorber of the RFB was ~10 mm. The transducer was excited with a continuous wave (CW) signal, and with a 50% duty cycle (DC) sine wave burst using a Keysight 33220A (Keysight, Santa Rosa, CA, USA) signal generator. The signal to the transducer was amplified with an E&A 2100L (Electronics and Innovation LTD, Rochester, NY, USA) power amplifier.

The peak-to-peak signal amplitude setting of the signal generator was varied on the following scale of values: Vin = [20 40 60 80 100 120 140 150 160 180 200] mVpp. The continuous-type signal was set to sine wave. The 50% DC burst sine wave was set to T_{ON} = 100 µs, T_{OFF} = 100 µs, PRF = 5 kHz.

For each measurement point, a total of 5 ON / 5 OFF cycles with an approximate period of 5s were recorder with the RFB and the total average measured power ($\overline{P_{OUT}}$) was calculated as:

$$\overline{P_{OUT}} = \frac{\sum_1^5 \overline{P_{ON}} - \overline{P_{OFF}}}{5}$$

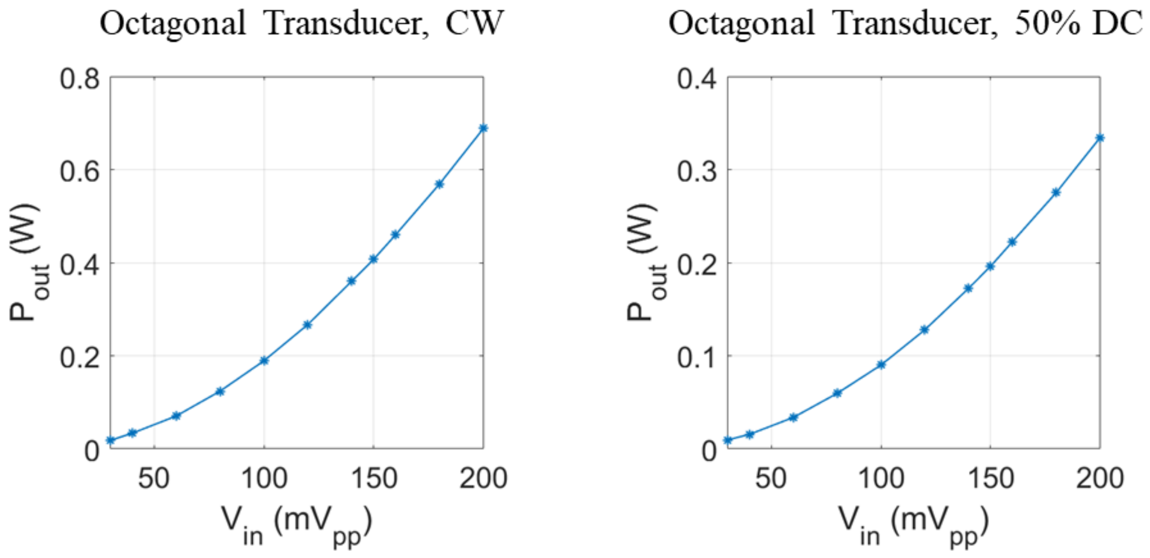

Figure S3: Correlation of mVpp to pressure amplitude using the RFB calibration.

Beam profile measurements

Beam profiling of the acoustic field from the single element transducer was performed with a three degree of freedom (3-DOF) linear scanner attached to a 0.5 mm diameter needle hydrophone (NH) (Precision Acoustics Ltd, Dorchester, United Kingdom). The pressure-sensitivity data was calculated from the NH readings via a complex (magnitude-phase) sensitivity deconvolution method described in [1].

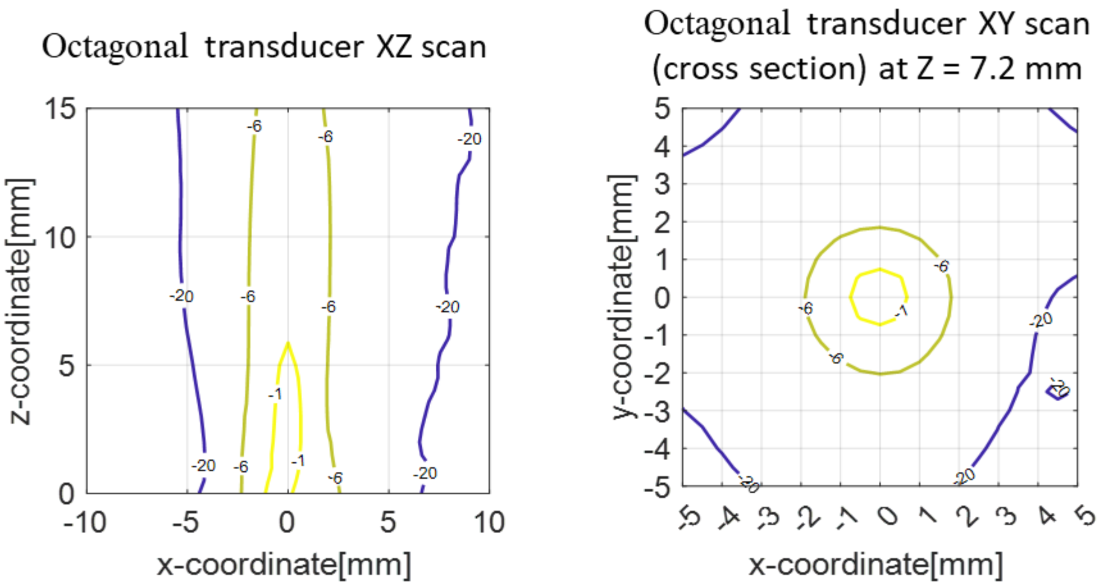

Figure S4: The image depicts the XZ scans of the symmetric and asymmetric transducers.

Table S1: The table shows the beam average diameter of the transducers after the XZ scan.

	Beam average diameter (mm)
Octagonal transducer XZ scan	3.9
Octagonal transducer XZ scan	4

Ultrasound intensity calculation

The intensity spatially averaged over the area enclosed by the half-pressure-maximum contour (ISAL) is calculated as [2]:

$$I_{SAL} = 0.867 * \frac{P}{D^2}$$

Where P is the total average power calculated $P = \overline{P_{OUT}}$; and D is the -6dB beam diameter. Note, we used the diameter measured from the XY cross section scans in the calculations of ISAL.

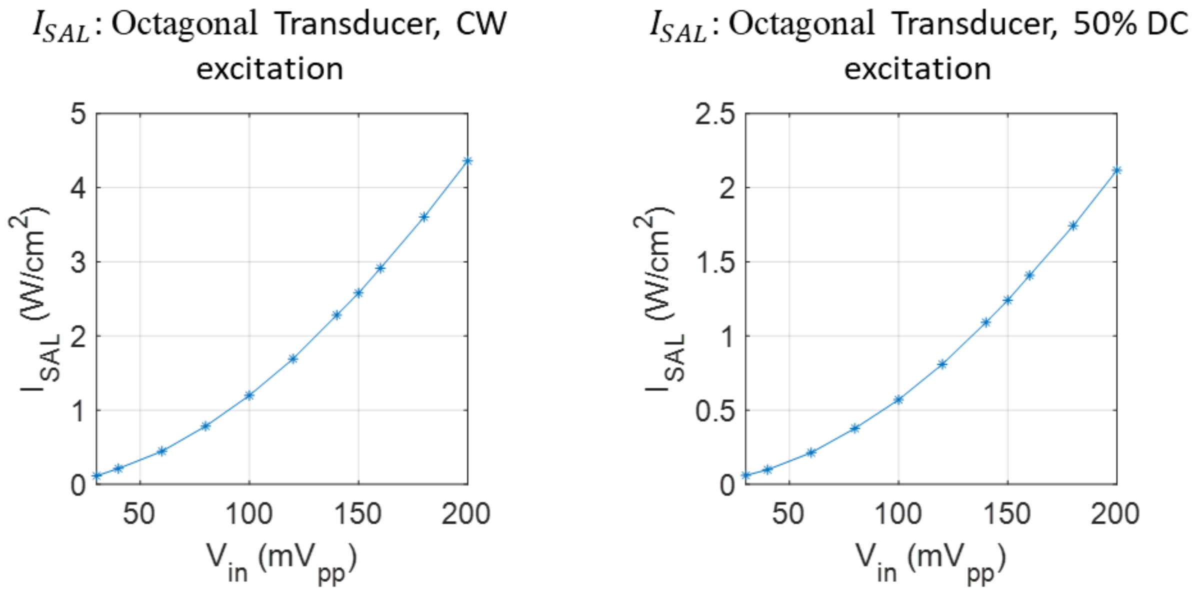

Figure S5: The figure depicts the intensity spatially averaged over the area enclosed by the half-pressure-maximum contour (I_{SAL})

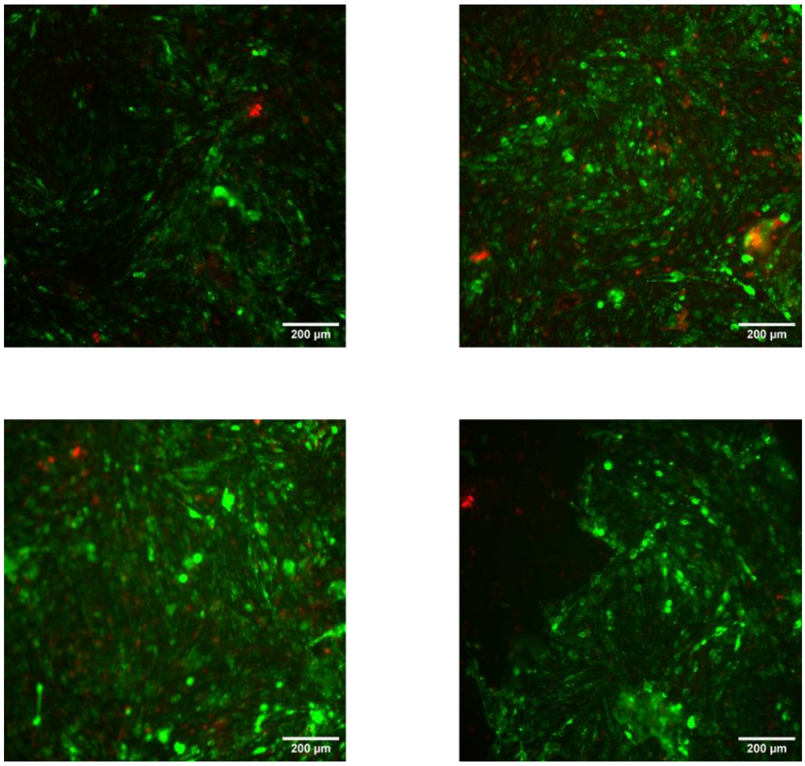

Figure S6: In this figure we observe various combined images of the well plates where the cell death and hole formation by the ND cavitation compromise the integrity of the BBB model.

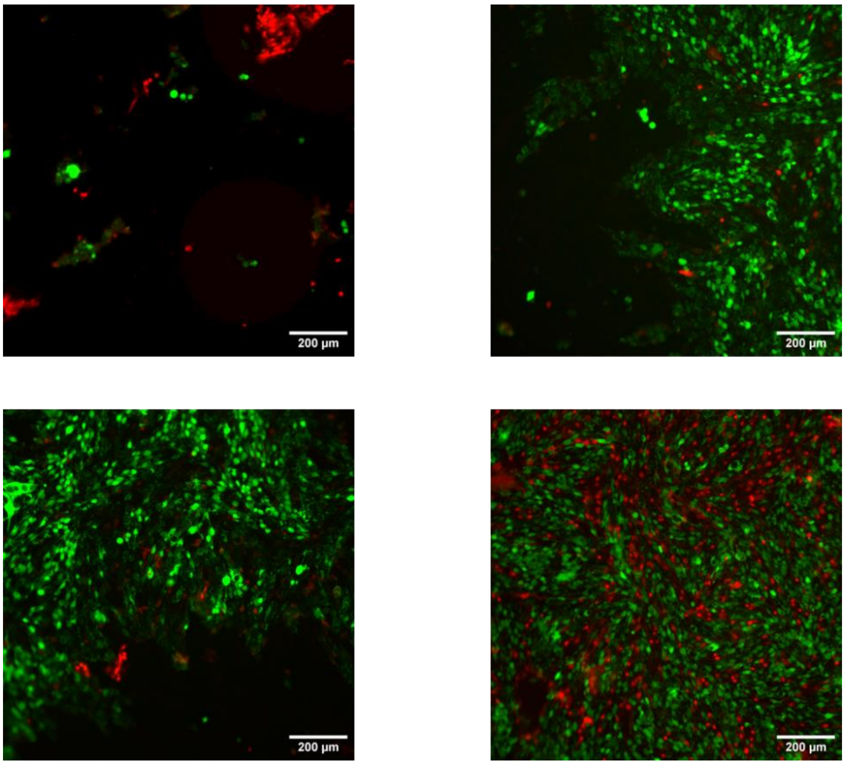

Figure S7: In this figure we observe various combined images of the well plates where the cell death and hole formation by the MB cavitation compromise the integrity of the BBB model in a more violent way.

Video S1: With High-Speed Camera (HSC) imaging, we have observed the cavitation dynamics exhibited by Sonazoid ® microbubbles (MBs) when subjected to ultrasound at a frequency of 1.1 MHz, accompanied by a peak negative pressure of 0.60 MPa. The video illustrates the expansion and subsequent collapse of the MBs in synchrony with the ultrasonic frequency.

https://emckclac-my.sharepoint.com/:v:/g/personal/k1927326_kcl_ac_uk/Edkd60DZ0QhLIhuzNyv_Cf4BMUuK5UQvitNU9MUW8wSajg?e=5Saty3

Video S2: With High-Speed Camera (HSC) imaging, we have observed the cavitation dynamics exhibited by PFP NDs when subjected to ultrasound at a frequency of 1.1 MHz, accompanied by a peak negative pressure of 0.60 MPa. The video illustrates the expansion and subsequent collapse of the NDs in synchrony with the ultrasonic frequency.

https://emckclac-my.sharepoint.com/:v:/g/personal/k1927326_kcl_ac_uk/EcWrRjQrunxMqFyj8ZpD_zwBx0vPktCyu-iihAnDhte_-Q?e=u20baE

Video S3: With High-Speed Camera (HSC) imaging, we have observed the cavitation dynamics exhibited by PFH NDs when subjected to ultrasound at a frequency of 1.1 MHz, accompanied by a peak negative pressure of 0.60 MPa. The video illustrates the expansion and subsequent collapse of the NDs in synchrony with the ultrasonic frequency.

https://emckclac-my.sharepoint.com/:v:/g/personal/k1927326_kcl_ac_uk/EfWxjUEBXxhAgIHmRGuP0ZAB8RnmneW6n-bd_glilNr8RQ?e=plSpyy

Video S4: With High-Speed Camera (HSC) imaging, we have observed the cavitation dynamics exhibited by PFP NDs when subjected to ultrasound at a frequency of 1.1 MHz, accompanied by a peak negative pressure of 0.12 MPa. The video illustrates the expansion and subsequent collapse of the NDs in synchrony with the ultrasonic frequency.

https://emckclac-my.sharepoint.com/:v:/g/personal/k1927326_kcl_ac_uk/ERP9lYwQyypNouB-fnT3aUsB_ySA1pQWKUSai_q1wBoBqg?e=74kyKf
